# HEMGN and SLC2A1 might be potential diagnostic biomarkers of steroid-induced osteonecrosis of femoral head: study based on WGCNA and DEGs screening

**DOI:** 10.1186/s12891-021-03958-7

**Published:** 2021-01-15

**Authors:** Zhixin Wu, Yinxian Wen, Guanlan Fan, Hangyuan He, Siqi Zhou, Liaobin Chen

**Affiliations:** 1 Department of Orthopedic Surgery, Zhongnan Hospital of Wuhan University, 169 Donghu Road, Wuhan City, 430071 Hubei Province China; 2 Department of Obstetrics and Gynecology, Zhongnan Hospital of Wuhan University, Wuhan, Hubei China

**Keywords:** Steroid-induced osteonecrosis of the femoral head, Weighted gene correlation network analysis, Differentially expressed genes, Diagnostic biomarkers, Peripheral blood

## Abstract

**Background:**

Steroid-induced osteonecrosis of the femoral head (SONFH) is a chronic and crippling bone disease. This study aims to reveal novel diagnostic biomarkers of SONFH.

**Methods:**

The GSE123568 dataset based on peripheral blood samples from 10 healthy individuals and 30 SONFH patients was used for weighted gene co-expression network analysis (WGCNA) and differentially expressed genes (DEGs) screening. The genes in the module related to SONFH and the DEGs were extracted for Gene Ontology (GO) and Kyoto Encyclopedia of Genes and Genomes (KEGG) pathway enrichment analysis. Genes with |gene significance| > 0.7 and |module membership| > 0.8 were selected as hub genes in modules. The DEGs with the degree of connectivity ≥5 were chosen as hub genes in DEGs. Subsequently, the overlapping genes of hub genes in modules and hub genes in DEGs were selected as key genes for SONFH. And then, the key genes were verified in another dataset, and the diagnostic value of key genes was evaluated by receiver operating characteristic (ROC) curve.

**Results:**

Nine gene co-expression modules were constructed via WGCNA. The brown module with 1258 genes was most significantly correlated with SONFH and was identified as the key module for SONFH. The results of functional enrichment analysis showed that the genes in the key module were mainly enriched in the inflammatory response, apoptotic process and osteoclast differentiation. A total of 91 genes were identified as hub genes in the key module. Besides, 145 DEGs were identified by DEGs screening and 26 genes were identified as hub genes of DEGs. Overlapping genes of hub genes in the key module and hub genes in DEGs, including *RHAG*, *RNF14*, *HEMGN*, and *SLC2A1*, were further selected as key genes for SONFH. The diagnostic value of these key genes for SONFH was confirmed by ROC curve. The validation results of these key genes in GSE26316 dataset showed that only *HEMGN* and *SLC2A1* were downregulated in the SONFH group, suggesting that they were more likely to be diagnostic biomarkers of SOFNH than *RHAG* and *RNF14*.

**Conclusions:**

Our study identified that two key genes, *HEMGN* and *SLC2A1*, might be potential diagnostic biomarkers of SONFH.

**Supplementary Information:**

The online version contains supplementary material available at 10.1186/s12891-021-03958-7.

## Background

Steroid-induced osteonecrosis of the femoral head (SONFH) is a chronic and crippling disease of the femoral head, due to the disruption of the blood supply of the femoral head and the subsequent death of bone cells after chronic exposure to excessive glucocorticoids, which ultimately results in the collapse of the femoral head and dysfunction of the hip joint [1]. There are approximately 10,000 to 20,000 new non-traumatic osteonecrosis of the femoral head (NONFH) cases reported each year in the United States alone, while the estimated NONFH cases in China were 8.12 million in the population aged 15 years and over, among which SONFH counted for 47.4% of the total NONFH cases [2–4]. Although the diagnostic criteria for SONFH have been established, SONFH patients are often diagnosed at the advanced stage (ARCO stage III-IV), due to the non-specific clinical symptoms at the early stage and the absence of specific biomarkers for the early diagnosis, which often leads to total hip arthroplasty of the patients [5]. Therefore, it is exceptionally urgent to establish novel early diagnostic molecular markers for SONFH.

Many studies have identified the biomarkers of diseases by screening differentially expressed genes (DEGs). It is worth noting that few studies have explored clusters of highly correlated genes, which may play key a role in clinical features of interests. Weighted gene co-expression network analysis (WGCNA), a particularly useful systems biology method in this context, helps to construct free-scale gene co-expression networks and detect gene modules [6]. By analyzing the connectivity between modules and clinical features, we can determine which modules are associated with which features. Notably, WGCNA has been widely used to identify the hub genes related to clinical features in different diseases, such as osteoarthritis [7], acute myocardial infarction [8], bladder cancer [9] and pancreatic cancer [10].

**Fig. 1:**
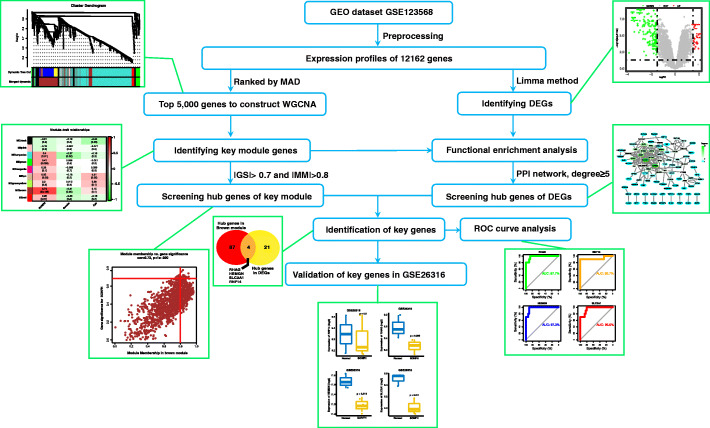
Flowchart used in the present study. GEO, Gene Expression Omnibus; MAD, median absolute deviation; WGCNA, weighted gene co-expression network analysis; DEGs, differentially expressed genes; PPI, protein-protein interaction; abs, absolute value; GS, gene significance; MM, module membership; ROC, receiver operating characteristic

In the present study, we proposed to identify novel diagnostic biomarkers of SONFH based on WGCNA and DEGs screening on the basis of GSE123568 dataset. The flowchart used in the present study was presented in Fig. 1. The key module with the highest level of significant correlation with SONFH was identified, and the genes with |gene significance| > 0.7 and |module membership| > 0.8 were then selected as hub genes in the key module. DEGs were integrated into a protein-protein interaction network analysis using the STRING database and Cytoscape software to identify hub genes (degree≥5). The overlapped genes between hub genes in the key module and hub genes in DEGs were defined as key genes (*RHAG*, *RNF14*, *HEMGN* and *SLC2A1*), and then the key genes were validated in GSE26316 dataset. This study may provide novel insight into the underlying mechanisms of SONFH and contribute to identifying potential diagnostic biomarkers of SONFH.

## Methods

### Data collection and preprocessing

The GSE123568 dataset was downloaded from the GEO database [11]. The gene annotation platform of the GSE123568 dataset was GPL15207 (Affymetrix Human Gene Expression Array). The GSE123568 dataset consisted of 10 peripheral blood samples from healthy individuals and 30 peripheral blood samples from SONFH patients (Table S1). The RAW data from the GSE123568 dataset were preprocessed using the R package “affy” and normalized using the “rma” method. Then, the data were filtered using the R package “genefilter” and the “nsFilter” method. Subsequently, probes were annotated using the R package “annotate”. To ensure the reliability of the annotation, probe sets mapped to > 1 gene were removed. In cases where a gene corresponds to a plurality of probe sets, the maximum value was used as the gene expression value. Eventually 40 samples and 12,162 genes were processed for subsequent analysis.

### WGCNA

After ranking the genes according to the median absolute deviation from largest to smallest, we selected the top 5000 genes for WGCNA using the R package “WGCNA” [6, 12]. Subsequently, we screened out the power parameter ranging from 1 to 20 using the “pickSoftThreshold” (package WGCNA) function. A suitable soft threshold of 4 was selected because it is the minimum power value that satisfied the degree of independence of 0.95. And then we obtained 9 modules through dynamic branch cutting with 0.25 as the merging threshold. Furthermore, based on the difference of the Topological Overlap Matrix and their cluster dendrogram, we visualized the resulting gene network as a heatmap.

### Identification of key modules

To identify key modules that were significantly related to clinical features, the correlation between the module feature genes and clinical features was analyzed. The correlation values were shown in a heatmap. The module most significantly associated with the SONFH statue was considered as the key module of SONFH. Gene significance (GS) referred to the correlation between gene expression and each trait, while module membership (MM) referred to the correlation between gene expression and each module eigengene. To verify specific module-trait associations, we examined the correlation between GS and MM. All correlation analyses in this study were carried out by using the Pearson correlation described in the ‘WGCNA’ package [6]. The genes with |GS| > 0.7 and |MM| > 0.8 were then selected as hub genes in the key module.

### DEGs screening

The “limma” package from R [13] was used to screen for the DEGs between healthy individuals and SONFH patients. The adjusted *P* value < 0.05 and |log2(fold change)| > 1.5 were used as the cut-off thresholds for the GSE123568 dataset. The heatmap of DEGs was plotted with R package “pheatmap”.

### Protein-protein interaction (PPI) network analysis and key genes screening

PPI network can help us identify the hub genes in DEGs between healthy individuals and SONFH patients. PPI information of DEGs was acquired from the Search Tool for the Retrieval of Interacting Genes (STRING) database. Then, Cytoscape software (v3.7.1) was used for the construction of PPI network. Hub genes in DEGs were identified as genes that had a ≥ 5 degree of connectivity. The overlapping genes of hub genes in the brown module and hub genes in DEGs were defined as key genes, which were significantly correlated with SONFH and easily affected by SONFH.

### Functional enrichment analysis of key module

To assess the biological function of key module genes and DEGs, the Gene Ontology (GO) functional terms and Kyoto Encyclopedia of Genes and Genomes (KEGG) pathways enrichment analysis were performed using Database for Annotation, Visualization, and Integrated Discovery (DAVID) tool (version 6.8) [14–16]. The GO terms and KEGG pathways with *p*-value < 0.05 and count > 1 were considered significant. For the genes in the key module, the top 10 GO terms and KEGG pathways functional enrichment ranked by gene count were visualized. For the DEGs between healthy individuals and SONFH patients, all significant GO terms and KEGG pathways functional enrichment were visualized.

### Validation of key genes in a public database

To validate these key genes in an external dataset, GEO was searched with the keywords “femoral head necrosis”. The inclusion criteria for the dataset used for validation were as follows: i) femoral head necrosis induced by steroid (glucocorticoid); ii) gene expression profiles; iii) the sample size of each group was ≥3. Based on these criteria, we identified 1 appropriate dataset, namely GSE26316, which included femoral heads from 3 normal rats and 3 SONFH rats. Subsequently, the key genes were assessed in the GSE26316 dataset for further validation. The expression profile from GSE26316 dataset was analyzed using the same method as described above for GSE123568.

### Receiver operating characteristic (ROC) curve analysis

To investigate the effect of gene expression on the disease state of SONFH, we plotted ROC curve based on the gene expression data and the status of the samples from the GSE123568 dataset. The ROC curves were plotted by the “pROC” package in R software [17]. The entropy weight method was used to determine the weight of each gene, and then the ROC curve of the four key genes was plotted. The diagnostic value of these four genes for SONFH was assessed using the area under the curve (AUC) obtained from the ROC curve analysis.

## Results

### Construction of WGCNA network

**Fig. 2:**
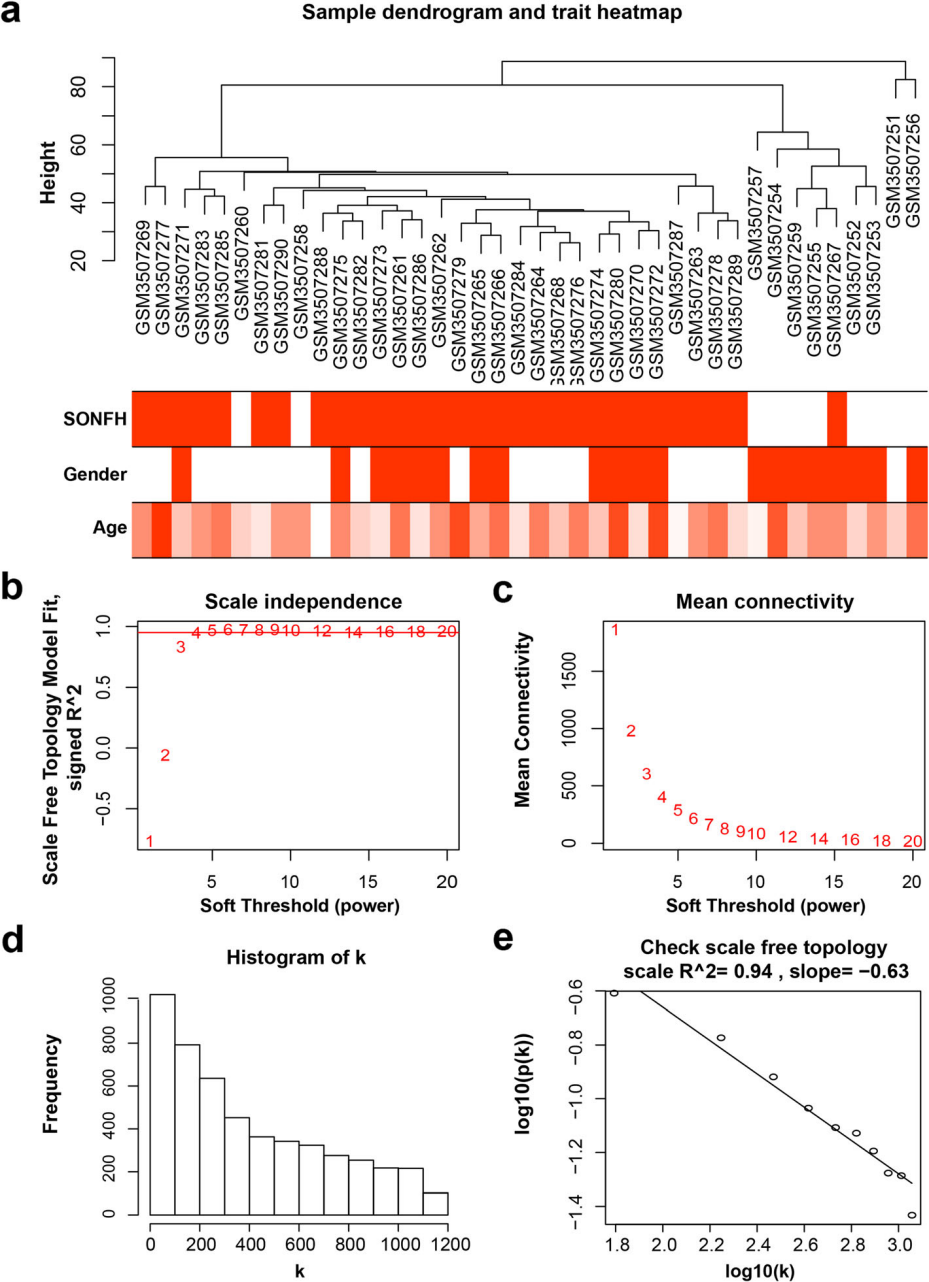
Sample clustering and soft threshold screening. **a** Sample clustering to detect outliers and the trait heatmap to display the sample traits. **b** Analysis of the scale-free fit index for various soft-thresholding powers (β). **c** Analysis of the mean connectivity for different soft-thresholding powers. **d** Histogram of the connectivity distribution when β = 4. **e** Verification of the scale-free topology when β = 4. WGCNA, weighted gene co-expression network analysis

After preprocessing and filtering for the GSE123568 dataset, we selected the top 5000 genes ranked by median absolute deviation from large to small for subsequent analysis. The results of sample clustering showed that there is no significant difference in the samples included in the WGCNA (Fig. 2a). When the power value (β) was set to 4, the scale independence value achieved 0.95 (Fig. 2b), and the co-expression networks met the requirements of scale-free topology (Fig. 2c, d). Thus, β = 4 was selected to produce a hierarchical clustering tree with different colors representing different modules.

### Identification and visualization of the key module

**Fig. 3:**
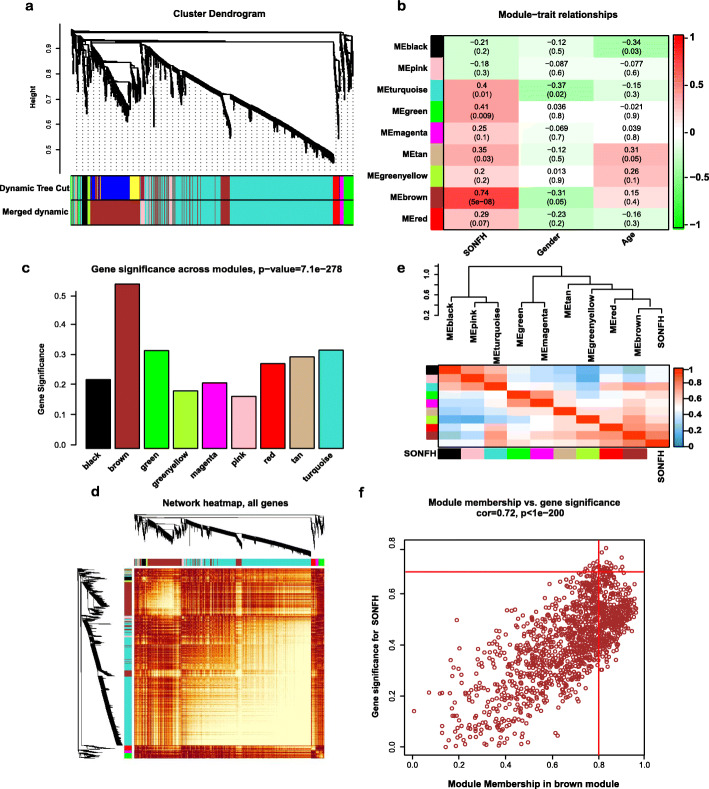
WGCNA of samples. **a** The cluster dendrogram of genes. **b** Module–trait relationships. Each cell consists of the corresponding correlation and *P*-value, which are color-coded by correlated according to the color legend. **c** Distribution of average gene significance and errors in the modules associated with SONFH status. **d** Visualizing all genes from the network using a heatmap plot to depict the TOM among the genes in the analysis. **e** The combination of eigengene dendrogram and heatmap. **f** A scatter plot of the GS for SONFH versus the MM in the brown module. WGCNA, weighted gene co-expression network analysis; TOM, topological overlap matrix; SONFH, steroid-induced osteonecrosis of the femoral head; GS, gene significance; MM, module membership

We then set power = 4 to produce a hierarchical clustering tree. Nine modules were identified with a merging threshold of 0.25 (Fig. 3a). Among all modules, the brown module with 1258 genes was the most relevant for SONFH status (Fig. 3b, c). All genes were selected for the heatmap (Fig. 3D). The eigengene dendrogram and heatmap were used to identify groups of correlated eigengenes. The result also indicated that the brown module was significantly associated with SONFH status (Fig. 3e). The GS for SONFH and MM in the brown module showed a strongly significant correlation, which indicated that genes in the brown module were highly correlated with SONFH (Fig. 3f). Subsequently, the genes with the |GS| > 0.7 and the |MM| > 0.8 were then selected as hub genes, and a total of 91 hub genes in the brown module were chosen for further analysis (Table S2).

### Functional enrichment analysis of brown module genes

**Fig. 4:**
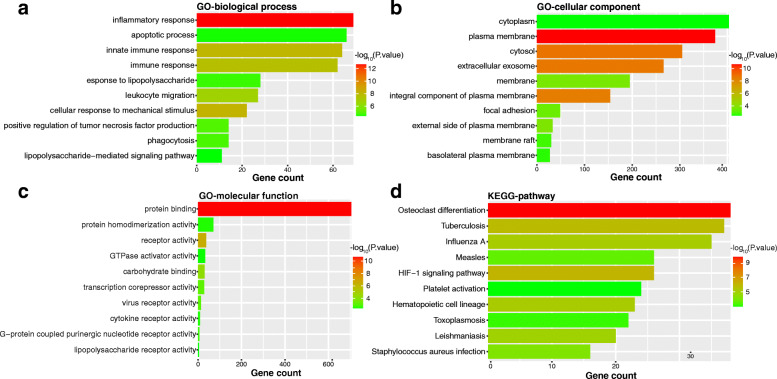
Functional enrichment analysis of genes related to SONFH. **a** The top 10 functional terms of GO-BP enrichment analysis. **b** The top 10 functional terms GO-CC enrichment analysis. **c** The top 10 functional terms GO-MF enrichment analysis. **d** The top 10 pathways of KEGG enrichment analysis. SONFH, steroid-induced osteonecrosis of the femoral head; GO, Gene Ontology; BP, biological process; CC, cellular component; MF, molecular function; KEGG, Kyoto Encyclopedia of Genes and Genomes

The genes in the brown module were used for GO analysis and KEGG pathway enrichment analysis to explore the underlying biological process correlated to SONFH. The top 10 significant GO terms and KEGG pathways functional enrichment ranked by gene count were visualized. For GO-biological process (GO-BP) enrichment analysis, the results showed that the genes involved in inflammatory response, apoptotic process, etc. were significantly enriched (Fig. 4a). For GO-cellular component (GO-CC) enrichment analysis, the genes involved in cytoplasm, plasma membrane, etc. were significantly enriched (Fig. 4b). For GO-molecular function (GO-MF) enrichment analysis, the genes involved in protein binding, protein homodimerization activity, etc. were significantly enriched (Fig. 4c). The results of the KEGG pathway analysis showed that the genes involved in osteoclast differentiation, tuberculosis, etc. were significantly enriched (Fig. 4d).

### Identification and analysis of DEGs

**Fig. 5:**
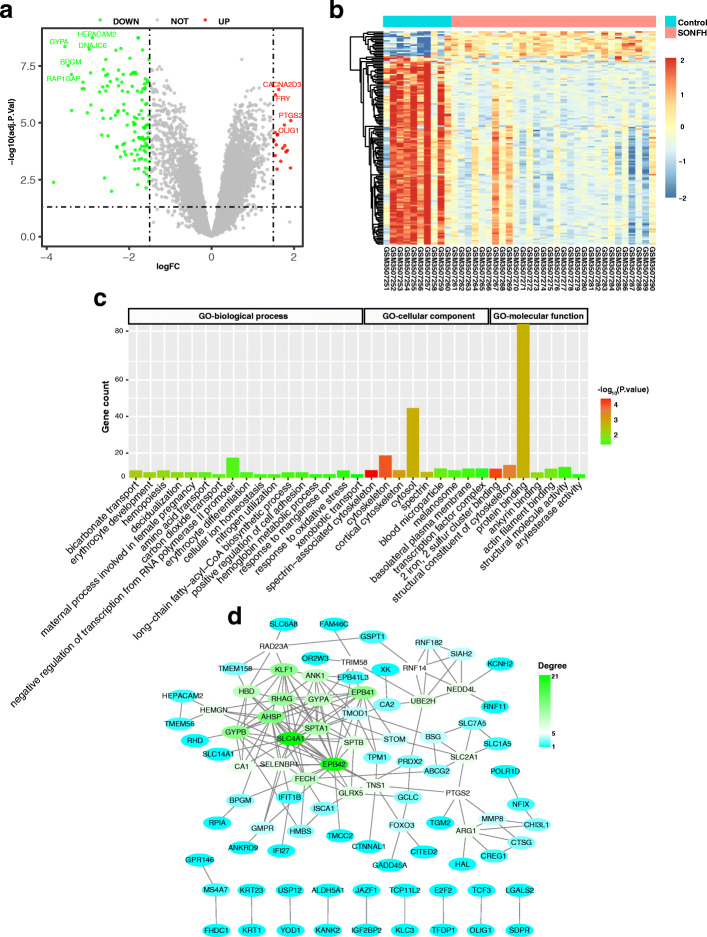
Analysis of DEGs. **a**, **b** Volcano and heatmap of DEGs between healthy individuals and SONFH patients. The adjusted *P*-value < 0.05 and |log2(fold change| > 1.5 and were used as the cut-off threshold. **c** The GO-BP, GO-CC and GO-MF functional terms identified after functional enrichment analysis of the DEGs. **d** PPI network analysis of DEGs. DEGs, differentially expressed genes; SONFH, steroid-induced osteonecrosis of the femoral head; GO, Gene Ontology; BP, biological process; CC, cellular component; MF, molecular function; PPI, protein-protein interaction network

Subsequently, we screened DEGs between the peripheral blood of healthy individuals and SONFH patients on the basis of GSE123568 dataset. One hundred forty-five DEGs were screened out, including 17 upregulated genes and 128 downregulated genes (Fig. 5a, b, and Table S2). We further performed GO and KEGG enrichment analysis for DEGs. The results of GO enrichment analysis showed that DEGs were mainly involved in negative regulation of transcription from RNA polymerase II promoter, cytosol, cytoskeleton and protein binding, with KEGG pathways enrichment analysis showing no significant enrichment (Fig. 5c). The DEGs with the degree of connectivity ≥5 were then selected as hub genes, of which, 26 hub genes in DEGs were chosen for further analysis (Fig. 5d, Table S4).

### Identification and verification of key genes

**Fig. 6:**
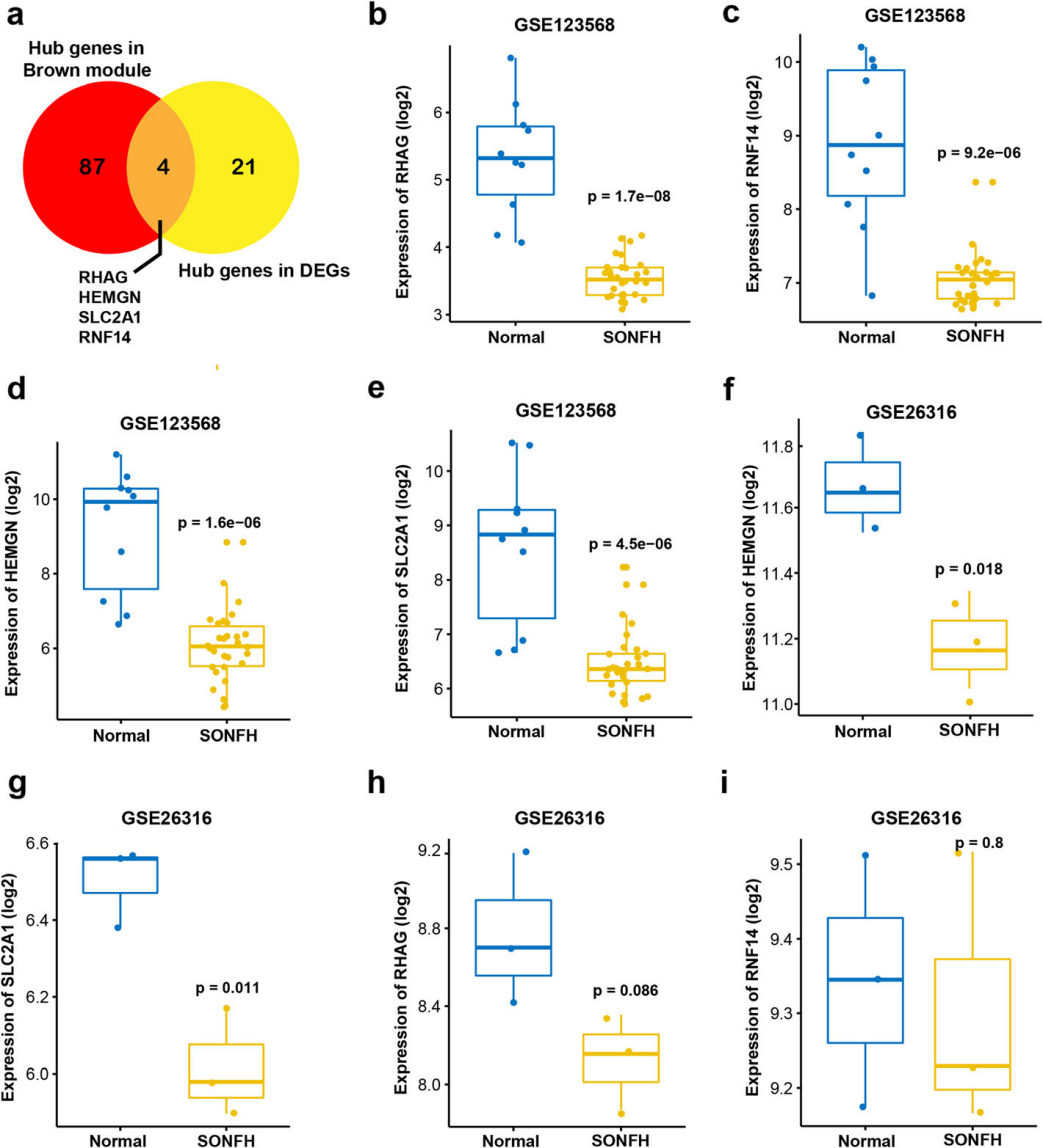
Analysis of key genes. **a** The Venn diagram of hub genes in the brown module and hub genes in DEGs. **b**-**e** Expression of *RHAG*, *RNF14*, *HEMGN*, *SLC2A1* in the GSE123568 data set. **f**-**i** Expression of *RHAG*, *RNF14*, *HEMGN*, *SLC2A1* in the GSE26316 dataset. *P* < 0.05 is considered statistically significant

Among the 26 hub genes in DEGs, *RHAG, RNF14, HEMGN and SLC2A1* were identified as the key genes for SONFH status and were selected for subsequent analysis (Fig. 6a, Table S4). We further visualized the expression of *RHAG, RNF14, HEMGN and SLC2A1* in the GSE123568 dataset and found that the expression of these four genes was significantly lower in SONFH group compared with the normal group (*P <* 0.05, Fig. 6b-e). Subsequently, the 4 genes were verified in another dataset GSE26316, and the results showed that the expression levels of *HEMGN* and *SLC2A1* were lower in the SONFH group (*P* < 0.05, Fig. 6f, g). However, no statistical difference in the gene expression of *RHAG and RNF14* was obtained (Fig. 6h, i).

### ROC curve analysis of key genes

**Fig. 7:**
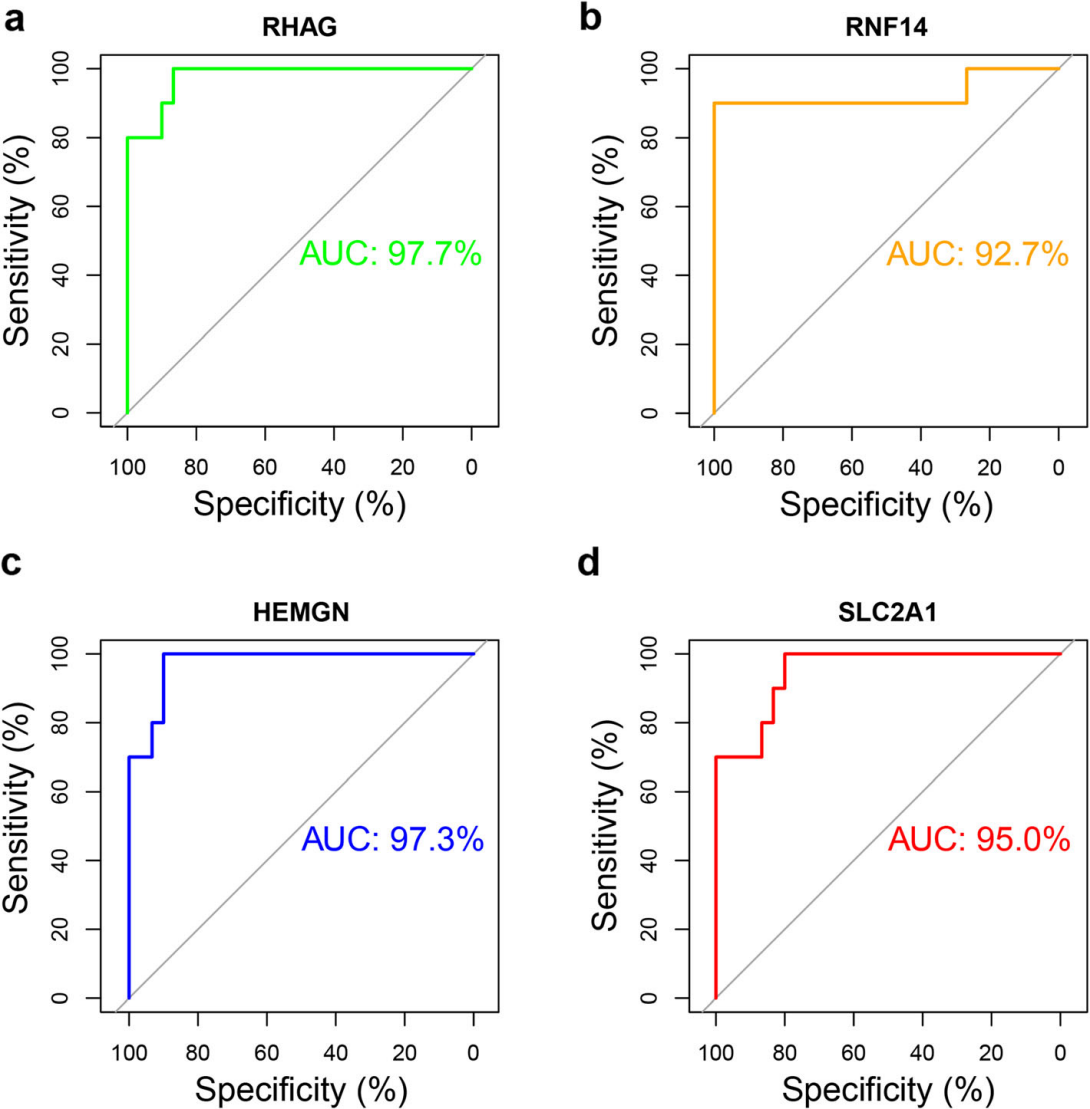
The ROC curve of key genes in SONFH. **a** *RHAG*. **b** *RNF14*. **c** *HEMGN*. **d** *SLC2A1*. The *x*-axis shows specificity, and the *y*-axis shows sensitivity. ROC, receiver operating characteristic; AUC: area under the ROC curve

Based on the *RHAG*, *RNF14*, *HEMGN* and *SLC2A1* expression data in GSE123568 and the disease state of the samples, the ROC curve was plotted to assess the diagnostic value of these four genes for SONFH. As shown in Fig. 7, the AUC of these genes were all > 0.9, which indicated that these genes might serve as potential diagnostic markers for SONFH.

## Discussion

SONFH is a complex disorder of the hip, of which the pathogenesis is multifactorial, with genetic and environmental factors playing a role [18]. Clinically, SONFH is sometimes difficult to be predicted or diagnosed because it is often asymptomatic at the early stage. Therefore, the femoral heads of some patients have already collapsed at the onset of the symptoms. Although total hip arthroplasty is considered as a definitive therapy of femoral head osteonecrosis, SONFH patients still face the psychological and economic burdens of revision surgery, as SONFH patients are often quite young when they received total hip arthroplasty [19]. Therefore, it is necessary to identify novel biomarkers for the early diagnosis of SONFH and the subsequent procedures for joint preservation.

The advancement of genomics and proteomics contribute to significant advances in the diagnosing and treating of many rare diseases and pathological conditions [20]. The biochip of GSE123568 consists of 10 peripheral blood samples from healthy individuals and 30 peripheral blood samples from SONFH patients. In the current study, the WGCNA algorithm and DEGs screening method were adopted to identify SONFH diagnostic biomarkers based on GSE123568. We found that the module with 1258 genes was the most relevant to SONFH status. The key module genes were significantly enriched in inflammatory response, apoptotic process and osteoclast differentiation. In fact, inflammation has been shown to play an essential role in the occurrence of SONFH. Animal experiments already indicated that the pathogenesis of SONFH involves inflammatory macrophage polarization mediated by pro-inflammatory tumor necrosis factor-α (TNF-α) [21]. The administration of anti-inflammatory IFN-β could inhibit IL-6 secretion and suppress osteonecrosis [22]. Additionally, the death of bone cell was found to be an essential pathogenic process of SONFH [23, 24]. Moreover, it is well known that abnormal osteoclast activity could lead to loss of bone structural integrity and subchondral fracture in osteonecrosis of the femoral head. A recent study has found that osteoclast numbers were elevated in rat or human femoral head of SONFH, which ultimately resulted in bone loss in the femoral heads and collapse of the femoral head [25]. To sum up, the key module genes were found to be significantly enriched in the inflammatory response, apoptotic process and osteoclast differentiation during the pathogenesis of SONFH.

Notably, 145 DEGs with 26 hub genes were screened out between peripheral blood samples of healthy individuals and SONFH patients, and the DEGs were mainly involved in negative regulation of transcription from RNA polymerase II promoter. Then, the intersection genes of hub genes in the key module and hub genes in DEGs were identified as the key genes for SONFH status, namely *RHAG, RNF14, HEMGN and SLC2A1.* To verify the results of bioinformatics analysis, we used the ROC curve to predict the diagnostic value of *RHAG, RNF14, HEMGN, SLC2A1*. The results showed that these genes might serve as diagnostic markers for SONFH because the AUC of these four genes was > 0.9.

*RHA*G, a member of the solute transporter family SLC42, is involved in the transportation of ammonia across erythrocyte membranes and appears to affect the function of erythrocyte in oxygen transport [26–28]. However, no existing study reported the role of *RHAG* in SONFH so far. *RNF14*, a human prostate coactivator, could induce androgen receptor (AR) target gene expression and regulate the AR signaling pathway [29]. Yang et al. [30] reported that the low expression of heterogeneous nuclear ribonucleoprotein A1 might activate *RNF14*-enhanced cell growth and contribute to prostate cancer progression. Wu et al. [31] reported that *RNF14* is a regulator of TCF/β-catenin to activate the Wnt pathway in colon cancer cells. These studies indicate that *RNF14* is an oncoprotein in several human cancers. However, whether *RNF14* is associated with SONFH remains unclear. *HEMGN*, also known as RP59 in *Rattus norvegicus*, is expressed in bone marrow cells and osteoblasts and participates in osteoblast recruitment [32, 33]. Jiang et al. [34] reported that *HEMGN* is a direct transcriptional target of HOXB4 and induces the expansion of myeloid progenitor cells. Thus, we speculate that *HEMGN* could participate in the occurrence of SONFH based on its involvement in the process of myeloid progenitor cell expansion and osteoblast recruitment. *SLC2A1*, a facilitative glucose transporter, is responsible for constitutive or basal glucose uptake [35, 36]. Chen et al. [37] showed that deletion of *SLC2A1* in osteoblast mostly blocked the bone anabolic function of Wnt7b in vivo, and impaired osteoblast differentiation and mineralization in vitro. Another study showed that miR-140-5p may contribute to the development femoral head osteonecrosis via ubiquitin proteasome system [38], while *SLC2A1* was a target of miR-140-5p [39]. In addition, the abolition of PI3K/AKT signaling induced by miR-186 may contribute to the pathogenesis of non-traumatic osteonecrosis [40], while miR-186 targets the 3′ UTR of Glut1 (*SLC2A1*) [41]. These studies suggest that *SLC2A1* may be related to the occurrence of SOFNH. Furthermore, these four genes were verified in the GSE26316 dataset, and only the expression of *HEMGN* and *SLC2A1* was reduced in the SONFH group. Taken together, among these four key genes, *HEMGN* and *SLC2A1* are more likely to participate in the occurrence of SOFNH and are more suitable as diagnostic biomarkers of SOFNH than RHAG and RNF14, which requires further experimental validation in the future.

There are several highlights in the current study. Firstly, few studies have focused on identifying diagnostic biomarkers of SONFH. An expression profile of peripheral serum samples from healthy individuals and SONFH patients could contribute to a comprehensive understanding of SONFH and identifying SONFH diagnostic biomarkers. Secondly, WGCNA has a particular advantage in processing gene expression datasets because it could analyze the connectivity between modules and clinical features. However, the current study also has a limitation. The potential diagnostic biomarkers were validated on a dataset from rats of SONFH rather than blood samples of real patients with this disease. The main cause is that the number of SONFH cases is decreasing due to the more and more standardized clinical use of steroid hormones. Therefore, we chosen to use the data set from rats of SONFH to verify the key genes. These potential diagnostic biomarkers remain to be validated using blood samples of real patients with SONFH in further studies.

## Conclusions

This study was the first study that used the WGCNA and DEGs screening method to identify key genes and diagnostic biomarkers for SONFH. *HEMGN* and *SLC2A1* from peripheral blood are closely related to the the occurrence of SOFNH, which might be the potential diagnostic biomarkers of SOFNH based on peripheral blood. Still, further studies are needed to validate the possibility of *HEMGN* and *SLC2A1* to be the biomarkers for SONFH.

## Supplementary Information

**Additional file 1: Table S1.**

**Additional file 2: Table S2.**

**Additional file 3: Table S3.**

**Additional file 4: Table S4.**

**Additional file 5: Table S5.**

## Data Availability

Publicly available datasets were analyzed in this study. This data can be obtained from the Gene Expression Omnibus (GEO) database (GSE123568, https://www.ncbi.nlm.nih.gov/geo/query/acc.cgi?acc=GSE123568 and GSE26316, https://www.ncbi.nlm.nih.gov/geo/query/acc.cgi?acc= GSE26316).

## Abbreviations

BP: Biological process
CC: Cellular component
DEGs: Differentially expressed genes
GEO: Gene expression omnibus
GO: Gene ontology
GS: Gene significance
KEGG: Kyoto encyclopedia of genes and genomes
MAD: Median absolute deviation
MF: Molecular function
MM: Module membership
PPI: Protein-protein interaction
SONFH: Steroid-induced osteonecrosis of the femoral head
ROC: Receiver operating characteristic
TOM: Topological overlap matrix
WGCNA: Weighted gene co-expression network analysis

## References

[1] Mont MA, Cherian JJ, Sierra RJ, Jones LC, Lieberman JR (2015). Nontraumatic osteonecrosis of the femoral head: where do we stand today? A ten-year update. J Bone Joint Surg Am.

[2] Barney J, Piuzzi NS, Akhondi H. Femoral Head Avascular Necrosis. StatPearls. https://www.ncbi.nlm.nih.gov/books/NBK546658/#article-76242.s14. Accessed 6 July 2020. 31536264

[3] Zhao DW, Yu M, Hu K, Wang W, Yang L, Wang BJ, Gao XH, Guo YM, Xu YQ, Wei YS (2015). Prevalence of nontraumatic osteonecrosis of the femoral head and its associated risk factors in the Chinese population: results from a nationally representative survey. Chin Med J.

[4] Ikeuchi K, Hasegawa Y, Seki T, Takegami Y, Amano T, Ishiguro N (2015). Epidemiology of nontraumatic osteonecrosis of the femoral head in Japan. Mod Rheumatol.

[5] Moya-Angeler J, Gianakos AL, Villa JC, Ni A, Lane JM (2015). Current concepts on osteonecrosis of the femoral head. World J Orthop.

[6] Langfelder P, Horvath S (2008). WGCNA: an R package for weighted correlation network analysis. BMC Bioinformatics..

[7] Zhu N, Zhang P, Du L, Hou J, Xu B (2020). Identification of key genes and expression profiles in osteoarthritis by co-expressed network analysis. Comput Biol Chem.

[8] Liu Z, Ma C, Gu J, Yu M (2019). Potential biomarkers of acute myocardial infarction based on weighted gene co-expression network analysis. Biomed Eng Online.

[9] Giulietti M, Occhipinti G, Righetti A, Bracci M, Conti A, Ruzzo A, Cerigioni E, Cacciamani T, Principato G, Piva F (2018). Emerging biomarkers in bladder Cancer identified by network analysis of Transcriptomic data. Front Oncol.

[10] Zhou YY, Chen LP, Zhang Y, Hu SK, Dong ZJ, Wu M, Chen QX, Zhuang ZZ, Du XJ (2019). Integrated transcriptomic analysis reveals hub genes involved in diagnosis and prognosis of pancreatic cancer. Mol Med.

[11] Barrett T, Wilhite SE, Ledoux P, Evangelista C, Kim IF, Tomashevsky M, Marshall KA, Phillippy KH, Sherman PM, Holko M (2013). NCBI GEO: archive for functional genomics data sets--update. Nucleic Acids Res.

[12] Zhang B, Horvath S (2005). A general framework for weighted gene co-expression network analysis. Stat Appl Genet Mol Biol.

[13] Ritchie ME, Phipson B, Wu D, Hu Y, Law CW, Shi W (2015). Smyth GK: limma powers differential expression analyses for RNA-sequencing and microarray studies. Nucleic Acids Res.

[14] Balakrishnan R, Harris MA, Huntley R, Van Auken K, Cherry JM (2013). A guide to best practices for Gene Ontology (GO) manual annotation. Database (Oxford).

[15] Huang da W, Sherman BT, Lempicki RA: Systematic and integrative analysis of large gene lists using DAVID bioinformatics resources. Nat Protoc 2009;4(1):44–57. 10.1038/nprot.2008.211 19131956

[16] Klukas C, Schreiber F (2007). Dynamic exploration and editing of KEGG pathway diagrams. Bioinformatics..

[17] Robin X, Turck N, Hainard A, Tiberti N, Lisacek F, Sanchez JC, Muller M (2011). pROC: an open-source package for R and S+ to analyze and compare ROC curves. BMC Bioinformatics.

[18] Chang C, Greenspan A, Gershwin ME (2020). The pathogenesis, diagnosis and clinical manifestations of steroid-induced osteonecrosis. J Autoimmun.

[19] Tripathy SK, Goyal T, Sen RK (2015). Management of femoral head osteonecrosis: current concepts. Indian J Orthop.

[20] Ganau L, Prisco L, Ligarotti GKI, Ambu R, Ganau M. Understanding the Pathological Basis of Neurological Diseases Through Diagnostic Platforms Based on Innovations in Biomedical Engineering: New Concepts and Theranostics Perspectives. Medicines. 2018;5(1):22. 10.3390/medicines5010022 PMC5874587 29495320

[21] Wu X, Xu W, Feng X, He Y, Liu X, Gao Y, Yang S, Shao Z, Yang C, Ye Z (2015). TNF-a mediated inflammatory macrophage polarization contributes to the pathogenesis of steroid-induced osteonecrosis in mice. Int J Immunopathol Pharmacol.

[22] Kim KM, Wagle S, Moon YJ, Wang SI, Park BH, Jang KY, Kim JR (2018). Interferon beta protects against avascular osteonecrosis through interleukin 6 inhibition and silent information regulator transcript-1 upregulation. Oncotarget..

[23] Calder JD, Buttery L, Revell PA, Pearse M, Polak JM (2004). Apoptosis--a significant cause of bone cell death in osteonecrosis of the femoral head. J Bone Joint Surg Br.

[24] Weinstein RS, Nicholas RW, Manolagas SC (2000). Apoptosis of osteocytes in glucocorticoid-induced osteonecrosis of the hip. J Clin Endocrinol Metab.

[25] Chen K, Liu Y, He J, Pavlos N, Wang C, Kenny J, Yuan J, Zhang Q, Xu J, He W (2020). Steroid-induced osteonecrosis of the femoral head reveals enhanced reactive oxygen species and hyperactive osteoclasts. Int J Biol Sci.

[26] Bruce LJ, Guizouarn H, Burton NM, Gabillat N, Poole J, Flatt JF, Brady RL, Borgese F, Delaunay J, Stewart GW (2009). The monovalent cation leak in overhydrated stomatocytic red blood cells results from amino acid substitutions in the Rh-associated glycoprotein. Blood..

[27] Stewart AK, Shmukler BE, Vandorpe DH, Rivera A, Heneghan JF, Li X, Hsu A, Karpatkin M, O'Neill AF, Bauer DE (2011). Loss-of-function and gain-of-function phenotypes of stomatocytosis mutant RhAG F65S. Am J Physiol Cell Physiol.

[28] Genetet S, Ripoche P, Picot J, Bigot S, Delaunay J, Armari-Alla C, Colin Y, Mouro-Chanteloup I (2012). Human RhAG ammonia channel is impaired by the Phe65Ser mutation in overhydrated stomatocytic red cells. Am J Physiol Cell Physiol..

[29] Kang HY, Yeh S, Fujimoto N, Chang C (1999). Cloning and characterization of human prostate coactivator ARA54, a novel protein that associates with the androgen receptor. J Biol Chem.

[30] Yang Z, Chang YJ, Miyamoto H, Yeh S, Yao JL, di Sant'Agnese PA, Tsai MY, Chang C (2007). Suppression of androgen receptor transactivation and prostate cancer cell growth by heterogeneous nuclear ribonucleoprotein A1 via interaction with androgen receptor coregulator ARA54. Endocrinology..

[31] Wu B, Piloto S, Zeng W, Hoverter NP, Schilling TF, Waterman ML (2013). Ring finger protein 14 is a new regulator of TCF/beta-catenin-mediated transcription and colon cancer cell survival. EMBO Rep.

[32] Kruger A, Ellerstrom C, Lundmark C, Christersson C, Wurtz T (2002). RP59, a marker for osteoblast recruitment, is also detected in primitive mesenchymal cells, erythroid cells, and megakaryocytes. Dev Dyn.

[33] Wurtz T, Kruger A, Christersson C, Lundmark C (2001). A new protein expressed in bone marrow cells and osteoblasts with implication in osteoblast recruitment. Exp Cell Res.

[34] Jiang J, Yu H, Shou Y, Neale G, Zhou S, Lu T, Sorrentino BP (2010). Hemgn is a direct transcriptional target of HOXB4 and induces expansion of murine myeloid progenitor cells. Blood..

[35] Lee EE, Ma J, Sacharidou A, Mi W, Salato VK, Nguyen N, Jiang Y, Pascual JM, North PE, Shaul PW (2015). A protein kinase C phosphorylation motif in GLUT1 affects glucose transport and is mutated in GLUT1 deficiency syndrome. Mol Cell.

[36] Kapoor K, Finer-Moore JS, Pedersen BP, Caboni L, Waight A, Hillig RC, Bringmann P, Heisler I, Muller T, Siebeneicher H (2016). Mechanism of inhibition of human glucose transporter GLUT1 is conserved between cytochalasin B and phenylalanine amides. Proc Natl Acad Sci U S A.

[37] Chen H, Ji X, Lee WC, Shi Y, Li B, Abel ED, Jiang D, Huang W, Long F (2019). Increased glycolysis mediates Wnt7b-induced bone formation. FASEB J.

[38] Chen N, Xiao B, Wang S, Wei B (2020). Bioinformatics analysis of microRNA linked to ubiquitin proteasome system in traumatic osteonecrosis of the femoral head. Medicine (Baltimore).

[39] Bai F, Yu Z, Gao X, Gong J, Fan L, Liu F (2019). Simvastatin induces breast cancer cell death through oxidative stress up-regulating miR-140-5p. Aging (Albany NY).

[40] Xu W, Li J, Tian H, Wang R, Feng Y, Tang J, Jia J (2019). MicroRNA186-5p mediates osteoblastic differentiation and cell viability by targeting CXCL13 in nontraumatic osteonecrosis. Mol Med Rep.

[41] Sun P, Hu JW, Xiong WJ, Mi J (2014). miR-186 regulates glycolysis through Glut1 during the formation of cancer-associated fibroblasts. Asian Pac J Cancer Prev.

