# Supplementary material for: HEMGN and SLC2A1 might be potential diagnostic biomarkers of steroid-induced osteonecrosis of femoral head: study based on WGCNA and DEGs screening
Source: BMC Musculoskelet Disord. 2021 Jan 15;22:85. doi: 10.1186/s12891-021-03958-7 (PMC7811219; doi:10.1186/s12891-021-03958-7)
Supplement: Supplementary file 1 — Additional file 1: Table S1. [file 12891_2021_3958_MOESM1_ESM.pdf]

**Table S1. The clinical features information of 10 healthy individuals and 30 SONFH patients from GSE123568 dataset.**

| Samples | Age (years) | Status | Gender | Samples | Age (years) | Status | Gender |
|---|---|---|---|---|---|---|---|
| GSM3507251 | 31 | non-SONFH | Female | GSM3507271 | 33 | SONFH | Male |
| GSM3507252 | 30 | non-SONFH | Male | GSM3507272 | 61 | SONFH | Male |
| GSM3507253 | 43 | non-SONFH | Male | GSM3507273 | 27 | SONFH | Male |
| GSM3507254 | 57 | non-SONFH | Male | GSM3507274 | 33 | SONFH | Male |
| GSM3507255 | 45 | non-SONFH | Male | GSM3507275 | 46 | SONFH | Male |
| GSM3507256 | 52 | non-SONFH | Male | GSM3507276 | 49 | SONFH | Female |
| GSM3507257 | 23 | non-SONFH | Male | GSM3507277 | 66 | SONFH | Female |
| GSM3507258 | 43 | non-SONFH | Female | GSM3507278 | 51 | SONFH | Female |
| GSM3507259 | 33 | non-SONFH | Male | GSM3507279 | 60 | SONFH | Female |
| GSM3507260 | 31 | non-SONFH | Female | GSM3507280 | 60 | SONFH | Male |
| GSM3507261 | 51 | SONFH | Male | GSM3507281 | 26 | SONFH | Female |
| GSM3507262 | 44 | SONFH | Male | GSM3507282 | 31 | SONFH | Female |
| GSM3507263 | 44 | SONFH | Female | GSM3507283 | 43 | SONFH | Female |
| GSM3507264 | 49 | SONFH | Female | GSM3507284 | 27 | SONFH | Female |
| GSM3507265 | 40 | SONFH | Male | GSM3507285 | 50 | SONFH | Female |
| GSM3507266 | 52 | SONFH | Male | GSM3507286 | 27 | SONFH | Male |
| GSM3507267 | 45 | SONFH | Male | GSM3507287 | 22 | SONFH | Female |
| GSM3507268 | 33 | SONFH | Female | GSM3507288 | 20 | SONFH | Female |
| GSM3507269 | 45 | SONFH | Female | GSM3507289 | 29 | SONFH | Female |
| GSM3507270 | 28 | SONFH | Male | GSM3507290 | 43 | SONFH | Female |

SONFH, steroid-induced osteonecrosis of the femoral head.
