# Supplementary material for: HEMGN and SLC2A1 might be potential diagnostic biomarkers of steroid-induced osteonecrosis of femoral head: study based on WGCNA and DEGs screening
Source: BMC Musculoskelet Disord. 2021 Jan 15;22:85. doi: 10.1186/s12891-021-03958-7 (PMC7811219; doi:10.1186/s12891-021-03958-7)
Supplement: Supplementary file 2 — Additional file 2: Table S2. [file 12891_2021_3958_MOESM2_ESM.pdf]

Table S1. The top 30 hub genes in the brown module related to SONFH.

Gene symbol	GS.SONFH	p.GS.SONFH	MMbrown	p.MMbrown
TCF3	-0.85090499	3.56E-12	-0.897837	4.14E-15
RHAG	-0.850080358	3.92E-12	-0.869087	3.55E-13
HEPACAM2	-0.848925893	4.49E-12	-0.847889	5.06E-12
PIP5K1B	-0.834185843	2.30E-11	-0.815637	1.45E-10
PNP	-0.833027674	2.60E-11	-0.904248	1.28E-15
DNAJC6	-0.829992024	3.55E-11	-0.844605	7.37E-12
CISD2	-0.827873437	4.41E-11	-0.845059	7.01E-12
RIOK3	-0.82053106	9.11E-11	-0.834699	2.18E-11
STOM	-0.820361018	9.26E-11	-0.814215	1.66E-10
DYRK3	-0.816186869	1.38E-10	-0.823126	7.08E-11
SEC62	-0.814826148	1.57E-10	-0.847388	5.37E-12
DYNLL1	-0.811443013	2.14E-10	-0.82319	7.03E-11
BPGM	-0.806122159	3.45E-10	-0.819436	1.01E-10
TFDP2	-0.805963099	3.50E-10	-0.89007	1.55E-14
TSPAN7	-0.801316658	5.26E-10	-0.827939	4.38E-11
RNF14	-0.797716828	7.15E-10	-0.847184	5.49E-12
TFDP1	-0.794964665	9.01E-10	-0.805347	3.70E-10
TRAK2	-0.792035288	1.15E-09	-0.850459	3.75E-12
KANK2	-0.79066872	1.28E-09	-0.830817	3.27E-11
RAP1GAP	-0.787957003	1.60E-09	-0.801596	5.13E-10
POLR1D	-0.787368947	1.67E-09	-0.861501	9.66E-13
REXO2	-0.784823303	2.05E-09	-0.842337	9.51E-12
ALDH5A1	-0.78313504	2.34E-09	-0.895137	6.63E-15
MAP4K5	-0.780588446	2.85E-09	-0.82817	4.28E-11
THOC7	-0.779960312	2.99E-09	-0.821928	7.96E-11

LSM12	-0.779067583	3.20E-09	-0.85588	1.95E-12
RUNX2	0.778846658	3.26E-09	0.8086636	2.75E-10
CTNNAL1	-0.77803103	3.47E-09	-0.820878	8.81E-11
KIAA0247	0.777677539	3.56E-09	0.9131631	2.16E-16
ZNRF1	-0.777419021	3.63E-09	-0.801576	5.14E-10

WGCNA, weighted gene co-expression network analysis; SONFH, steroid-induced osteonecrosis of the femoral head; GS.SONFH, gene significance for steroid-induced osteonecrosis of the femoral head; p.GS.SONFH, p value of gene significance for steroid-induced osteonecrosis of the femoral head; MMbrown, module membership in brown module; p.MMbrown, p value of module membership in brown module.
