# Supplementary material for: HEMGN and SLC2A1 might be potential diagnostic biomarkers of steroid-induced osteonecrosis of femoral head: study based on WGCNA and DEGs screening
Source: BMC Musculoskelet Disord. 2021 Jan 15;22:85. doi: 10.1186/s12891-021-03958-7 (PMC7811219; doi:10.1186/s12891-021-03958-7)
Supplement: Supplementary file 3 — Additional file 3: Table S3. [file 12891_2021_3958_MOESM3_ESM.pdf]

Table S2. The top 30 DEGs between peripheral blood of healthy individuals and SONFH patients from the GSE123568 dataset.

Gene symbol	logFC	AveExpr	adj.*P*.Val	Changes
HEPACAM2	-2.88693	4.164768	1.87E-09	down
TCF3	-1.76997	7.496223	1.87E-09	down
RHAG	-1.77633	3.98868	1.87E-09	down
GYPA	-3.5515	4.72229	4.56E-09	down
PIP5K1B	-2.1923	5.582068	5.52E-09	down
DNAJC6	-2.96152	3.248769	5.90E-09	down
PNP	-1.66784	8.502888	6.38E-09	down
CISD2	-2.31412	7.441126	6.61E-09	down
RIOK3	-1.97491	9.582583	1.49E-08	down
DYRK3	-2.56037	4.815681	1.68E-08	down
STOM	-1.51312	9.141459	1.68E-08	down
BPGM	-3.47292	6.78371	3.11E-08	down
TSPAN7	-2.61745	5.163598	4.60E-08	down
TSTA3	-2.24982	8.437211	4.94E-08	down
RNF14	-1.84448	7.499247	6.15E-08	down
C9orf40	-1.89843	5.032622	6.28E-08	down
TMCC2	-2.92938	7.114248	6.51E-08	down
TFDP1	-1.89263	8.695385	6.72E-08	down
NSUN3	-1.82166	6.614407	7.16E-08	down
KANK2	-2.51437	5.771078	7.48E-08	down
TRAK2	-1.95099	8.400213	7.54E-08	down
GADD45A	-2.13578	7.130317	7.61E-08	down
RAP1GAP	-3.39371	6.77606	7.61E-08	down
YOD1	-2.56248	7.574687	8.50E-08	down

POLR1D	-1.60089	8.283607	1.20E-07	down
ALDH5A1	-2.28764	5.79538	1.23E-07	down
REXO2	-1.53969	7.007634	1.42E-07	down
UBE2H	-2.45176	7.337455	1.57E-07	down
C14orf45	-3.11211	5.487415	1.68E-07	down
CTNNAL1	-2.16892	5.521397	1.68E-07	down

DEGs, differentially expressed genes; SONFH, steroid-induced osteonecrosis of the femoral head; logFC, log2(fold change); AveExpr, average expression; adj.*P*.Val, adjusted *P* value. Analysis based on R package "limma": adjusted *P* value < 0.05 and $|\log2(\text{fold change})| > 1.5$ were used as the cut-off thresholds.
