# Supplementary material for: HEMGN and SLC2A1 might be potential diagnostic biomarkers of steroid-induced osteonecrosis of femoral head: study based on WGCNA and DEGs screening
Source: BMC Musculoskelet Disord. 2021 Jan 15;22:85. doi: 10.1186/s12891-021-03958-7 (PMC7811219; doi:10.1186/s12891-021-03958-7)
Supplement: Supplementary file 4 — Additional file 4: Table S4. [file 12891_2021_3958_MOESM4_ESM.pdf]

**Table S3. The 26 hub genes in DEGs between peripheral blood of healthy individuals and SONFH patients from the GSE123568 data set.**

| Gene symbol | logFC | AveExpr | adj.*P*.Val | Changes | Degree |
|---|---|---|---|---|---|
| *SLC4A1* | -2.2011 | 10.03726 | 0.000321 | down | 21 |
| *EPB42* | -1.7672 | 9.360564 | 0.001043 | down | 20 |
| *AHSP* | -2.5074 | 8.890725 | 3.83E-05 | down | 13 |
| *GYPB* | -3.00653 | 8.152934 | 3.72E-06 | down | 12 |
| *EPB41* | -1.55716 | 9.018125 | 1.25E-05 | down | 12 |
| *KLF1* | -2.14741 | 7.717545 | 9.39E-05 | down | 12 |
| *RHAG* | -1.77633 | 3.98868 | 1.87E-09 | down | 11 |
| *SPTA1* | -1.75053 | 3.613585 | 4.39E-06 | down | 10 |
| *ANK1* | -2.10273 | 7.343524 | 8.17E-05 | down | 9 |
| *HBD* | -2.29087 | 10.7805 | 0.000113 | down | 9 |
| *FECH* | -2.71042 | 8.737562 | 4.50E-07 | down | 9 |
| *GYPA* | -3.5515 | 4.72229 | 4.56E-09 | down | 9 |
| *SPTB* | -1.88625 | 7.416942 | 6.15E-05 | down | 7 |
| *GLRX5* | -1.75955 | 10.53396 | 4.54E-05 | down | 7 |
| *UBE2H* | -2.45176 | 7.337455 | 1.57E-07 | down | 7 |
| *ARG1* | -1.58242 | 4.986036 | 0.007486 | down | 6 |
| *NEDD4L* | -1.93304 | 5.848987 | 0.000239 | down | 6 |
| *SLC2A1* | -2.12363 | 7.003557 | 1.57E-06 | down | 6 |
| *CA1* | -3.38781 | 9.414098 | 2.96E-06 | down | 6 |
| *SELENBP1* | -2.07284 | 9.596969 | 0.000261 | down | 6 |
| *HEMGN* | -3.0837 | 6.84676 | 3.27E-07 | down | 6 |
| *TNS1* | -1.59561 | 8.145497 | 0.000997 | down | 6 |
| *RNF14* | -1.84448 | 7.499247 | 6.15E-08 | down | 5 |
| *PTGS2* | 1.927072 | 6.345205 | 8.18E-06 | up | 5 |

| | | | | | |
|---|---|---|---|---|---|
| *TRIM58* | -1.86284 | 9.297499 | 0.000467 | down | 5 |
| *RAD23A* | -1.61517 | 8.54838 | 1.75E-05 | down | 5 |

DEGs, differentially expressed genes; SONFH, steroid-induced osteonecrosis of the femoral head. logFC, log2(Fold Change); AveExpr, average expression; adj.P.Val, adjusted P value. Analysis based on R package "limma": adj.P.Val<0.05 and log2(fold change) were used as the cut-off thresholds for the GSE123568 dataset.
