# Supplementary material for: HEMGN and SLC2A1 might be potential diagnostic biomarkers of steroid-induced osteonecrosis of femoral head: study based on WGCNA and DEGs screening
Source: BMC Musculoskelet Disord. 2021 Jan 15;22:85. doi: 10.1186/s12891-021-03958-7 (PMC7811219; doi:10.1186/s12891-021-03958-7)
Supplement: Supplementary file 5 — Additional file 5: Table S5. [file 12891_2021_3958_MOESM5_ESM.pdf]

Table S4. Identification of key genes by comparing the hub genes in the brown module with the hub genes in DEGs using a Venn diagram web-tool.

Names	Total	Elements
Overlapping genes	4	*RHAG*, *HEMGN*, *SLC2A1*, *RNF14*
Hub genes in the brown module	87	*DNAJC6*, *ACSL6*, *TRAK2*, *TBCEL*, *SNN*, *SEC62*, *LSM12*, *CARHSP1*, *POLR1D*, *ATG16L2*, *RGS2*, *MAP4K5*, *MAP3K5*, *MXD4*, *BOD1L*, *CISD2*, *TCF3*, *USP12*, *ARNTL*, *MPPE1*, *VTI1B*, *ICAM3*, *CEP63*, *KANK2*, *ARHGAP25*, *CYB5A*, *ABCG2*, *BPGM*, *DPEP2*, *BICD2*, *THOC7*, *ALDH5A1*, *TIMM23*, *CTSS*, *ABCB10*, *CAMK2G*, *TACC3*, *SLC7A5*, *C14orf45*, *RHD*, *PXN*, *SIGLEC7*, *FAM117A*, *DYNLL1*, *DYRK3*, *RAP1GAP*, *FAM104B*, *ELOVL6*, *DCK*, *KBTBD11*, *RIOK3*, *PIK3CD*, *RASSF2*, *STOM*, *TFDP2*, *ATP6V1A*, *TFRC*, *CTNNAL1*, *CBL*, *TFDP1*, *UROD*, *TNFAIP2*, *REXO2*, *ZNRF1*, *TSPAN7*, *HMBS*, *RUNX2*, *PNP*, *DPCD*, *MYO1F*, *IFIT1B*, *UNKL*, *RERE*, *PIP5K1B*, *SGK1*, *DDI2*, *FAM174A*, *APLP2*, *FRY*, *KIAA0247*, *HEPACAM2*, *PGM2L1*, *ST8SIA4*, *GCAT*, *RPS6KA5*, *TMCC1*, *ISCA1*
Hub genes in DEGs	21	*EPB42*, *RAD23A*, *SPTA1*, *ANK1*, *FECH*, *SLC4A1*, *TNS1*, *KLF1*, *SELENBP1*, *AHSP*, *PTGS2*, *ARG1*, *EPB41*, *HBD*, *TRIM58*, *GLRX5*, *GYPB*, *NEDD4L*, *SPTB*, *CA1*, *UBE2H*
